# Overall survival among patients with activated phosphoinositide 3-kinase delta syndrome (APDS)

**DOI:** 10.1186/s13023-025-03734-z

**Published:** 2025-05-03

**Authors:** Malena Mahendran, Julia E. M. Upton, Ramya Ramasubramanian, Heidi L. Memmott, Guillaume Germain, Katharina Büsch, François Laliberté, Amanda Harrington

**Affiliations:** 1 Groupe d’analyse, Ltée, Montréal, Québec Canada; 2 Clinical Immunology and Allergy, Department of Pediatrics, The Hospital For Sick Children, Toronto, ON Canada; 3 Department of Pediatrics, University of Toronto, Toronto, ON Canada; 4 Pharming Healthcare, Inc, Warren, NJ USA; 5 KJM Büsch Consulting GmbH, Büsch, Switzerland

**Keywords:** Activated phosphoinositide 3-kinase delta syndrome (APDS), Hematopoietic stem cell transplant (HSCT), Inborn error of immunity (IEI), Lymphoma, Overall survival, Primary immunodeficiency (PID)

## Abstract

**Background:**

This study aimed to describe overall survival (OS) of patients with APDS relative to the global population as well as among subsets of patients with concurrent lymphoma or hematopoietic stem cell transplant (HSCT) relative to the overall APDS population.

**Methods:**

Patient-level data were extracted from a recent systematic literature review of 351 unique patients with APDS. OS was evaluated using the Kaplan-Meier method up to age 65 years. OS rate and corresponding 95% CI were reported at each decade of age. Global mortality estimates were obtained from World Health Organization life tables for 2019.

**Results:**

Of the 351 patients with APDS (APDS1, 267 [76.1%]; APDS2, 83 [23.6%]; unspecified, 1 [0.3%]), 41 (11.7%) died. The OS rate was 25.0% (95% CI, 1.6–62.7%) by the last death event at 64 years of age. Starting at 12 years of age, the OS rate was numerically lower in patients with APDS relative to the global population (median OS, 64 vs. 75 years, respectively). Relative to the overall APDS population, OS rates were numerically similar in those who underwent HSCT (median OS, 64 years for both; *p* = 0.569), whereas OS rates were numerically lower in patients with concurrent lymphoma (median OS, 41 vs. 64 years, respectively; *p* = 0.109). Publication bias in source data was a possible limitation.

**Conclusion:**

Reduced survival in patients with APDS suggests a high disease burden, particularly in those with concurrent lymphoma. These results highlight the unmet need for disease-modifying treatments for APDS.

## Introduction

Activated phosphoinositide 3-kinase delta (PI3Kδ) syndrome (APDS)—also known as p110 delta–activating variant causing senescent T cells, lymphadenopathy, and immunodeficiency (PASLI)—is an underrecognized, rare primary immunodeficiency (PID) that was first characterized in 2013 [1–3]. As an inborn error of immunity, APDS is caused by variants in one of two genes that encode subunits of PI3Kδ [3–5]. Gain-of-function mutations in the phosphatidylinositol-4,5-bisphosphate 3-kinase catalytic subunit delta (*PIK3CD*) gene encoding the p110δ catalytic subunit of PI3Kδ lead to APDS1, and loss-of-function mutations in the phosphoinositide-3-kinase regulatory subunit 1 (*PIK3R1*) gene encoding the p85α regulatory subunit of PI3Kδ lead to APDS2 [1, 2, 4–9]. These genetic mutations result in overactivation of the PI3K/AKT/mTOR/S6K signaling pathway, which alters B-cell and T-cell growth, survival, proliferation, and differentiation, ultimately leading to immune deficiency and dysregulation [4–6, 9, 10].

Patients diagnosed with APDS exhibit a diverse range of symptoms that typically present in infancy or early childhood, with APDS1 and APDS2 often displaying similar clinical features [4, 11, 12]. Recurrent respiratory tract infections are nearly ubiquitous among patients with APDS and may be accompanied by other manifestations such as bronchiectasis, persistent herpesvirus infections, various viral and bacterial infections, non-neoplastic lymphoproliferation involving lymphadenopathy, splenomegaly and hepatomegaly, autoimmune and autoinflammatory conditions, neurodevelopmental issues, and growth deficiencies [4, 11, 12]. Additionally, concurrent lymphoma has been reported in up to 25% of patients with APDS [11–13]. Although some adults with APDS may be asymptomatic, many patients experience considerable morbidity, and infection-related fatalities have been documented in children and young adults with APDS [11].

Manifestations associated with APDS are variable and may be progressive and detrimental over time [4]. There are currently no treatment guidelines defining standard of care. Therapies for APDS can vary according to clinical manifestations and may include antimicrobial prophylaxis, mTOR inhibitors, immunomodulatory therapies, immunoglobulin replacement therapy, and splenectomy [4]. Notably, none of these treatment strategies target the underlying pathogenesis of APDS. Hematopoietic stem cell transplant (HSCT) has been used to treat APDS when severe immune deficiency is present. However, the risks of adverse events and mortality limit the clinical application of HSCT [4, 14–16]. In 2023, leniolisib, an oral selective PI3Kδ inhibitor, became the first and only treatment approved in the US for APDS after meeting both coprimary outcomes of reduction in index lymph node size and increase in the percentage of naïve to total B cells in the peripheral blood (*p* < 0.001 for both).

As APDS was only characterized within the last decade, there is a paucity of published literature on the clinical course of the disease, including the survival pattern of patients with APDS [5, 6]. Small cohort studies of APDS1 and APDS2 have reported 30-year overall survival rates of 86% and < 75%, respectively [12, 16], while a larger systematic literature review of 256 patients with APDS reported a 30-year overall survival rate of 74% [17]. However, these analyses did not contextualize the overall survival of patients with APDS relative to the general population or account for the impact of therapies.

To shed light on these gaps in the literature, this study estimated the overall survival of patients with APDS relative to the global population using patient-level data from a systematic literature review. Additional objectives were to describe overall survival by APDS subtype, in patients with concurrent lymphoma, and censoring for patients who were treated with HSCT.

## Methods

Patient-level data were extracted via a systematic literature review that has been previously detailed [18]. Briefly, the systematic literature review followed the PICO (population, intervention, comparator, and outcome) principle (Table 1) [19]. A comprehensive literature search in PubMed and Embase databases was conducted from the time of each database’s inception to March 13, 2023, to identify relevant publications that included data on patients with APDS and their survival status [19, 20]. To be included in the study population, patients were required to have a reported APDS diagnosis *or* a first-degree relative with genetically confirmed APDS and at least 1 reported clinical sign consistent with APDS. When available, data on age at last observation, death, age at death, sex, APDS subtype (APDS1 or APDS2), concurrent lymphoma, HSCT, age at HSCT, and age at leniolisib initiation were extracted from the literature within the systematic literature review [1, 2, 7, 8, 11, 12, 15, 16, 21–120].

**Table 1:** PICO criteria

Category	Inclusion criteria	Exclusion criteria
Population	• Patients with APDS *OR* patients with ≥ 1 clinical sign consistent with the clinical spectrum of APDS^a^*AND* a first-degree relative who has a genetically confirmed diagnosis of APDS	• Studies not reporting individual patient data for outcome of interest
Interventions or comparators	• Any	• Not applicable
Outcomes	• Age at last observation • Alive status • Age at death	• No reported outcome of interest
Publication type	• Articles, letters, clinical communications, and case series	• Any other publications
Language	• English language • Study publication date: 2013^b^ to March 2023	• Studies published in languages other than English or prior to 2013^b^

^a^Clinical signs included documented severe recurrent sinopulmonary infections (> 2 events within 3 years of each other); bronchiectasis; lymphadenopathy for greater than 1 month; any nodular lymphoid hyperplasia; chronic hepatomegaly or chronic splenomegaly; severe, persistent, or recurrent Herpesviridae infections (e.g., Epstein-Barr virus, cytomegalovirus); autoimmune cytopenia; enteropathy; lymphoma; hypogammaglobulinemia; elevated levels of immunoglobulin M; reduced number of CD3^+^CD4^+^ T cells; increased number of follicular helper T cells; reduced number of naïve T cells; clinical diagnosis of CVID or a primary immunodeficiency; evidence of PI3K pathway activation; and additional clinical features within the clinical spectrum of APDS, with a consensus. ^b^Studies included were published in 2013 or later, as APDS was characterized in 2013. APDS, activated phosphoinositide 3-kinase delta syndrome; CVID, common variable immunodeficiency; PI3K, phosphoinositide 3-kinase; PICO, population, intervention, comparison, and outcome

Global mortality estimates were obtained from the World Health Organization (WHO) life tables for 2019 and were used to estimate overall survival for the global population [121].

### Statistical analysis

Overall survival was evaluated using the Kaplan-Meier method and defined as the time from birth to the age of death due to any cause. The overall survival rate and corresponding 95% CIs were estimated up to 65 years of age, as the maximum age of death reported in the individual patient data was 64 years. Median overall survival was defined as the age when the overall survival rate of patients with APDS was 50%. To ensure that the overall survival rate in patients with APDS accounted for therapies used as supportive care, patients treated with leniolisib were censored at the age of leniolisib initiation. In the analysis that censored for HSCT, patients who underwent HSCT were censored at the age of transplant.

The overall survival rate of the global population was derived using the probability of dying (*q*_*x*_) at specific age intervals. Overall survival rates of the global population were calculated using the following formula:

$$\:{S}_{t}=\left(1-{q}_{x\left(t\right)}\right)\:\times\:\:{S}_{t-1}$$

Where *S*_*t*_ represents the overall survival rate at age interval *t*, *q*_*x(t)*_ represents the probability of dying at time *t*, and *S*_*t–1*_ represents the overall survival rate at the prior age interval. The overall survival rate at 0 years of age was imputed as 1.

Overall survival rates between groups of patients were compared using a log-rank test to determine whether differences in survival were statistically significant. Comparisons were made between the following groups: (1) patients with APDS1 versus APDS2 (2) patients with APDS with concurrent lymphoma versus without concurrent lymphoma, and (3) patients with APDS who received HSCT versus those who did not receive HSCT.

All analyses were conducted using SAS Enterprise Guide software Version 7.15 (SAS Institute, Cary, NC).

## Results

### Patient characteristics

**Fig. 1:**
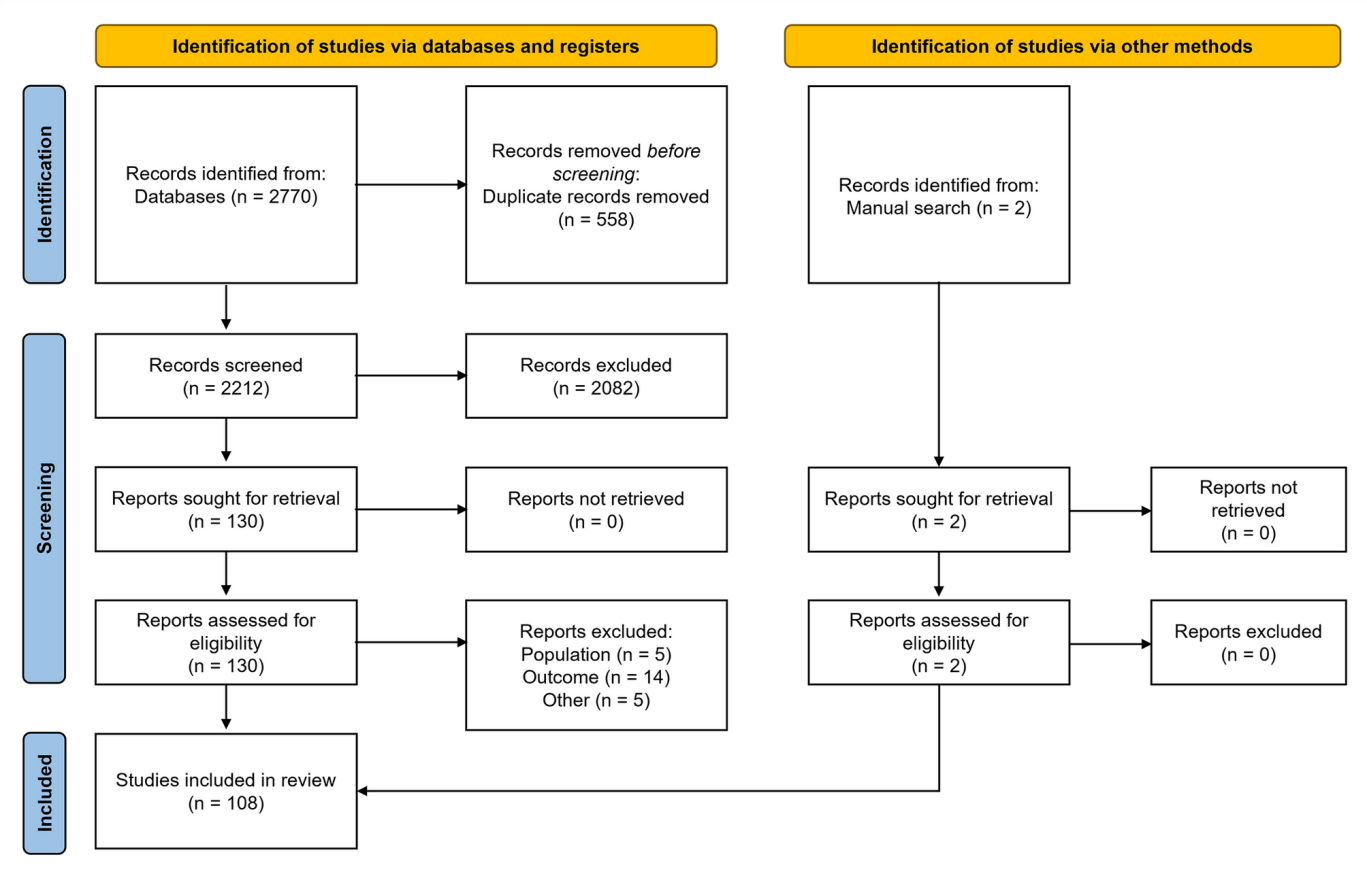
PRISMA flow diagram providing the review process. PRISMA, Preferred Reporting Items for Systematic Reviews and Meta-Analyses

Among 108 eligible publications from the systematic literature review (Fig. 1), 351 unique patients with APDS were identified: 267 patients (76.1%) with APDS1 and 83 patients (23.6%) with APDS2 (Table 2) [18]. One patient (0.3%) did not have a specified APDS subtype and was excluded from the stratified analyses. Overall, 171 patients (49%) were male, 135 (38%) were female, and 45 (13%) did not have sex reported. Lymphoma was reported in 43 patients (12.3%) with APDS. A total of 46 patients (13.1%) were reported to have undergone HSCT, of whom 6 patients were excluded from the censoring analysis, as the age at the time of the procedure was not reported. Leniolisib use was reported for 13 patients (3.7%), none of whom were treated with HSCT.

**Table 2:** Patient characteristics^a^

Characteristic	Patients (*N* = 351)
Sex, No. (%)	
Male	171 (49)
Female	135 (38)
Not reported	45 (13)
APDS type, No. (%)	
APDS1 (*PIK3CD*)	267 (76.1)
APDS2 (*PIK3R1*)	83 (23.6)
Not reported	1 (0.3)
Age at last follow-up	
Alive	
No. of patients with available data	310
Mean (range), y	17.1 (0.5–67)
Deceased	
No. of patients with available data	41
Mean (range), y	19.6 (1–64)

^a^Because of rounding, percentages may not total 100%. APDS, activated phosphoinositide 3-kinase delta syndrome; *PIK3CD*, phosphatidylinositol-4,5-bisphosphate 3-kinase catalytic subunit delta; *PIK3R1*, phosphoinositide-3-kinase regulatory subunit 1

### Overall survival

**Fig. 2:**
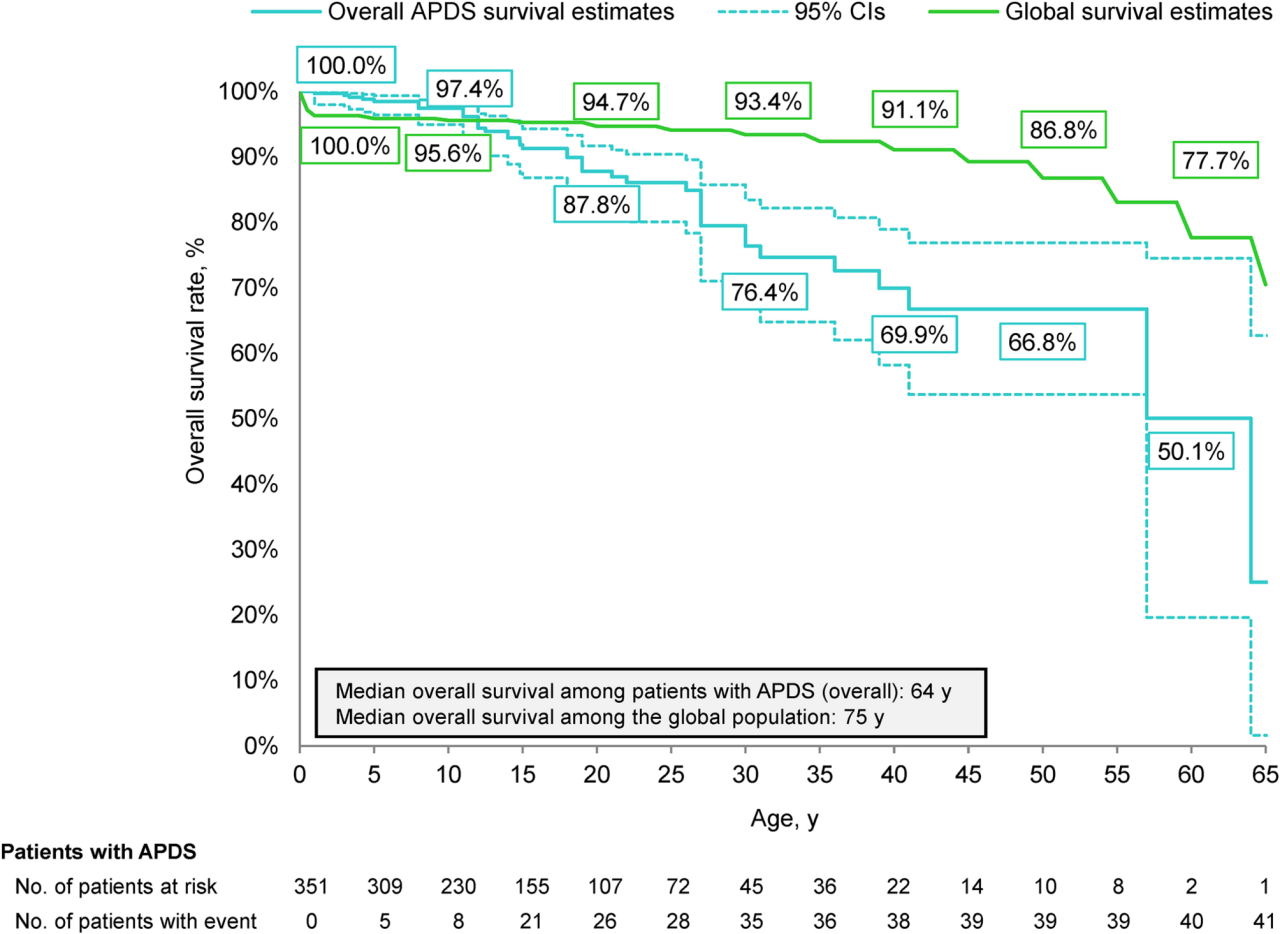
Kaplan-Meier curve of overall survival in patients with APDS and the global population. APDS, activated phosphoinositide 3-kinase delta syndrome

Of the 351 patients with APDS, 41 (11.7%) died, and the median overall survival was 64 years (Fig. 2). The estimated overall survival rate reached 25.0% (95% CI, 1.6–62.7%) by the last death event at 64 years of age. Starting from 12 years of age, the estimated overall survival of patients with APDS was numerically lower relative to the global population, with the estimated overall survival rate being 17.0% and 21.2% lower at 30 and 40 years of age, respectively. The median overall survival in the global population was 75 years.

**Fig. 3:**
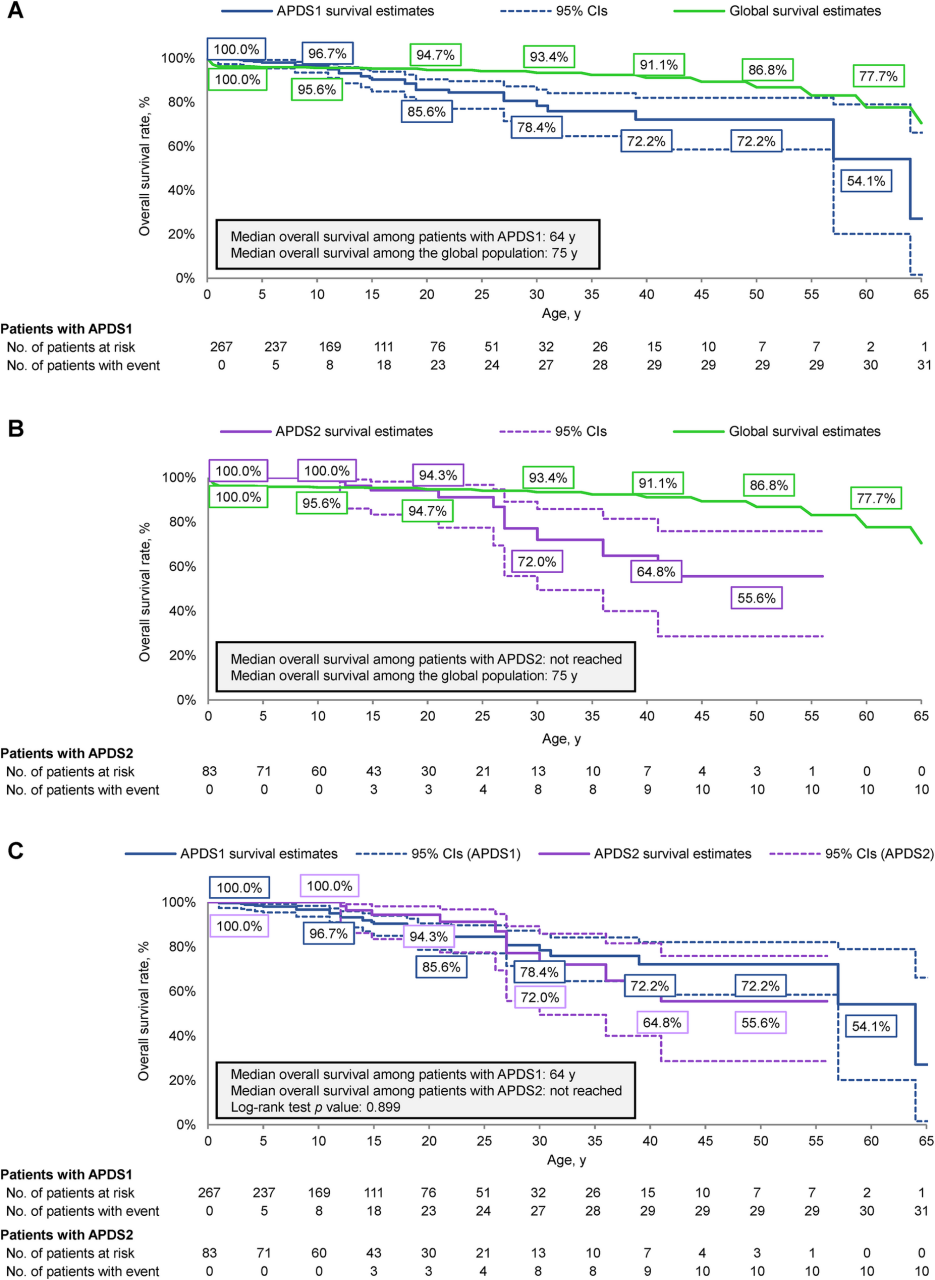
Kaplan-Meier curve of overall survival in patients with (**A**) APDS1 or (**B**) APDS2 and the global population, or (**C**) APDS1 versus APDS2. APDS, activated phosphoinositide 3-kinase delta syndrome

Among the 267 patients with APDS1, 31 (11.6%) died, and median overall survival was 64 years (Fig. 3A). The estimated overall survival rate among patients with APDS1 reached 27.1% (95% CI, 1.6–66.2%) by the last death event at 64 years of age. Starting from 11 years of age, the estimated overall survival of patients with APDS1 was numerically lower relative to the global population. At 30 and 40 years of age, the estimated overall survival rate was 15.0% and 18.9% lower, respectively, relative to the global population.

Among the 83 patients with APDS2, 10 (12.0%) died, and median overall survival was not reached (Fig. 3B). The estimated overall survival rate among patients with APDS2 reached 55.6% (95% CI, 28.6–75.9%) by the last death event at 41 years of age. Starting at nearly 15 years of age, the estimated overall survival of patients with APDS2 was numerically lower relative to the global population. At 30 and 40 years of age, the estimated overall survival rate was 21.4% and 26.3% lower, respectively, relative to the global population. There was no significant difference in overall survival between patients with APDS1 and patients with APDS2 (*p* = 0.899) (Fig. 3C).

**Fig. 4:**
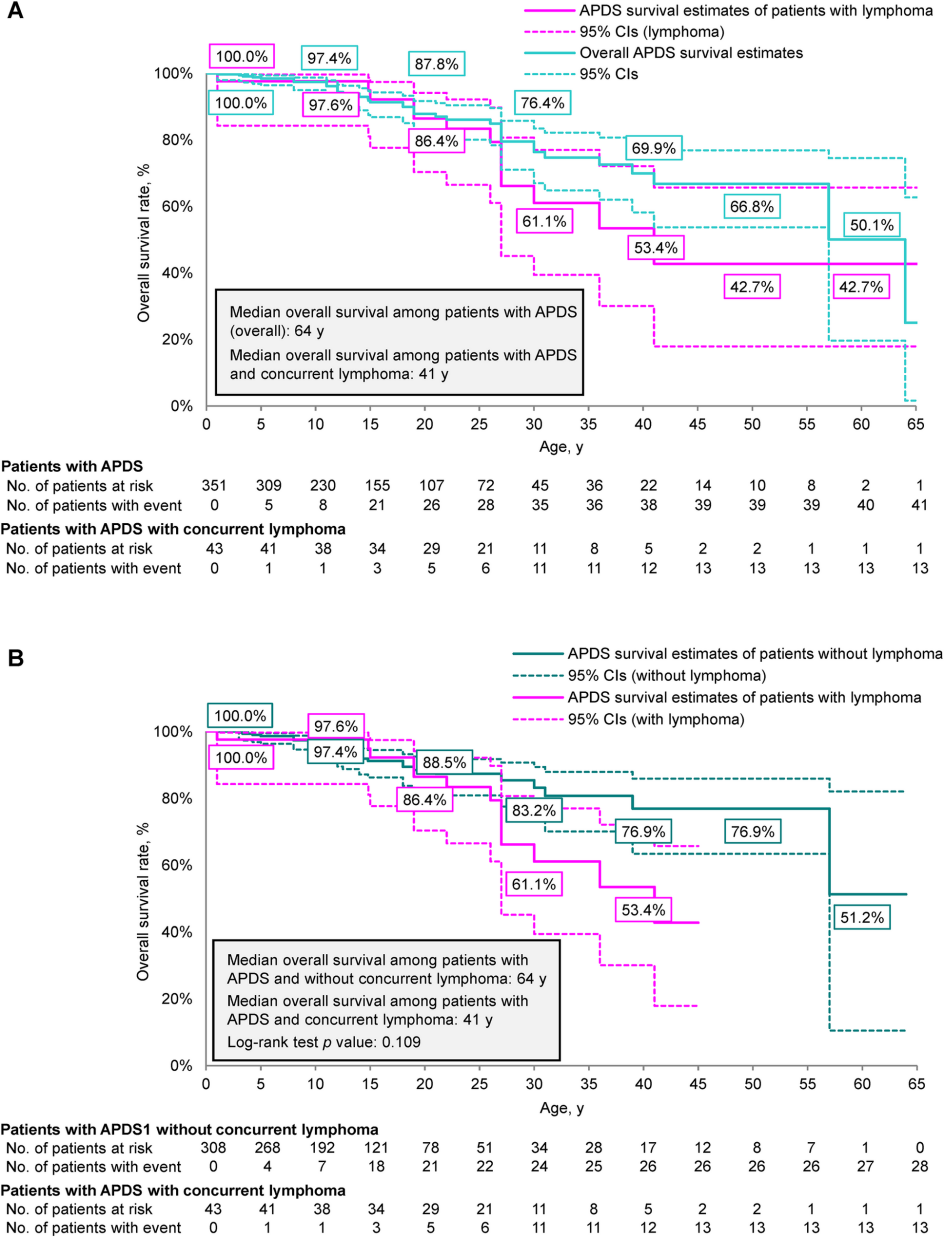
Kaplan-Meier curve of overall survival in (**A**) patients with APDS and APDS with concurrent lymphoma or (**B**) patients with APDS with concurrent lymphoma versus without concurrent lymphoma. APDS, activated phosphoinositide 3-kinase delta syndrome

In the 43 patients with APDS and reported concurrent lymphoma, 13 (30.2%) died and the median overall survival was 41 years (Fig. 4A). Relative to the overall population of patients with APDS, the estimated overall survival of patients with APDS and concurrent lymphoma was numerically lower across nearly all ages. The estimated overall survival rate among patients with APDS and concurrent lymphoma reached 42.7% (95% CI, 17.9–65.7%) by the last death event at 41 years of age, relative to the estimated overall survival rate of 66.8% (95% CI, 53.7–76.9%) in the overall population of patients with APDS at the same age. Overall survival was further compared between the 43 patients with concurrent lymphoma and the 308 patients without concurrent lymphoma (Fig. 4B). Although the overall survival rate was observed to be up to 23.5% lower in patients with APDS and concurrent lymphoma compared with those without lymphoma, the difference between these two groups was not significant (*p* = 0.109).

**Fig. 5:**
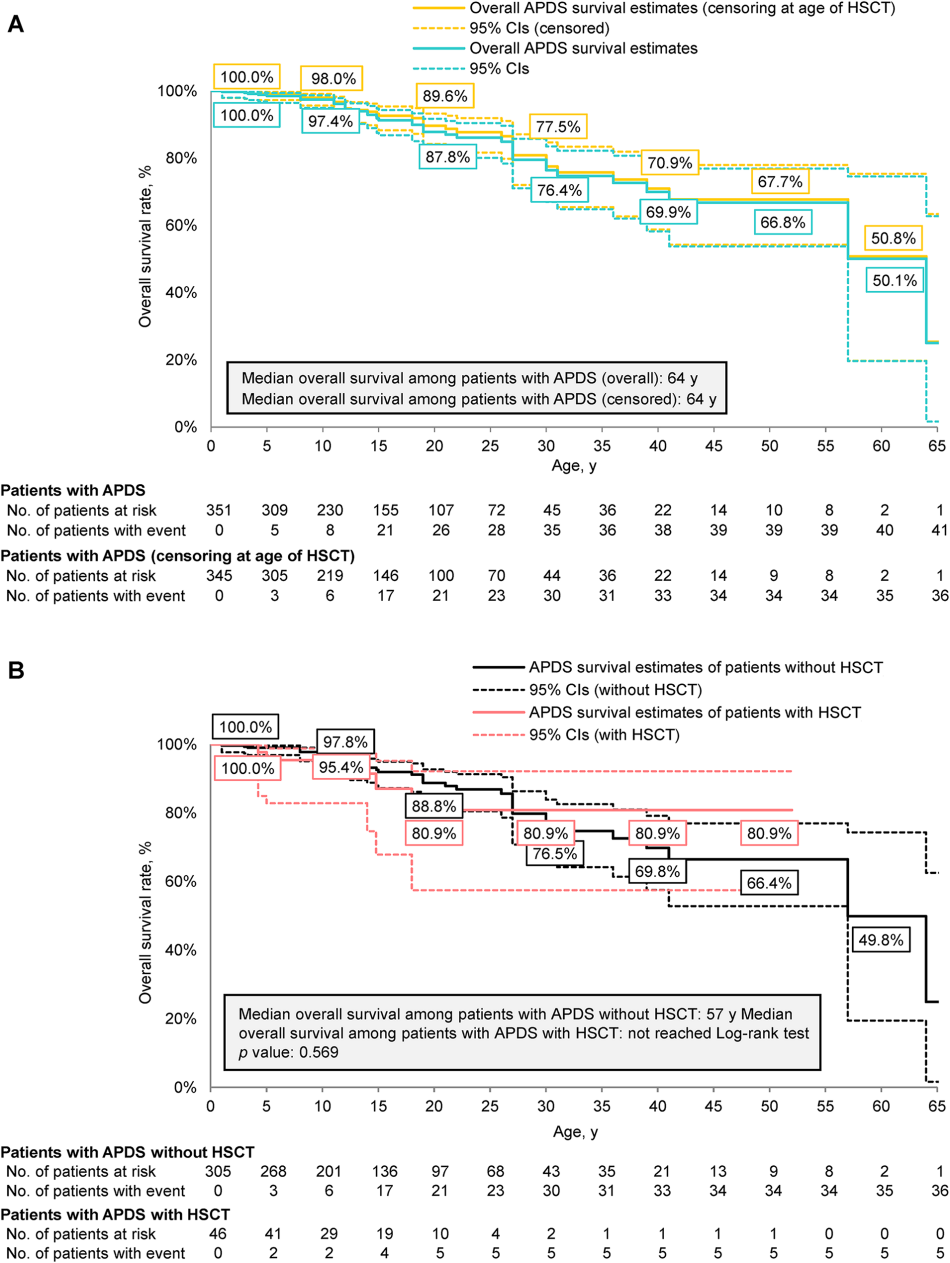
Kaplan-Meier curve of overall survival in (**A**) patients with APDS and censoring at age of HSCT or (**B**) patients with APDS with HSCT versus without HSCT. APDS, activated phosphoinositide 3-kinase delta syndrome; HSCT, hematopoietic stem cell transplant

Of 40 patients reported to have undergone HSCT and for whom an age at transplant was available, 5 (12.5%) died. The mean follow-up time between transplant and last reported age was 3.7 years (SD, 4.5; range, 0–16), with 16 (40%) and 9 (22.5%) patients having at least 3 and 5 years of follow-up after transplant, respectively. The overall survival of patients with APDS was largely consistent across ages after censoring for HSCT (Fig. 5A). The median overall survival of patients with APDS censored for HSCT was 64 years, consistent with the overall APDS population. Beginning at approximately 4 years of age, estimated overall survival when censoring for HSCT was numerically higher than the overall survival when not censoring for HSCT, with the largest difference in the estimated overall survival rate observed at 15 years of age (censored for HSCT, 92.3% [95% CI, 88.0–95.2%]; not censored for HSCT, 91.3% [95% CI, 86.8–94.3%]). Overall survival was also evaluated between the 46 patients with APDS who had undergone HSCT and those who had not (*n* = 305) (Fig. 5B). Although median overall survival among patients with APDS who had not undergone HSCT was 57 years and was not reached in those who had undergone HSCT, no significant difference was observed between these two groups (*p* = 0.569).

## Discussion

Using individual data from 351 patients with APDS obtained via a systematic literature review, this study estimated a lower overall survival rate among the overall APDS population relative to the global population and across APDS subtype. Early mortality in patients with APDS was evidenced by a median overall survival of 64 years, an 11-year difference compared with 75 years in the global population. In addition, overall survival rates for patients with APDS were up to 28% lower than that of the global population, underscoring the significant morbidity of APDS that can lead to shorter lifespans. The estimated 30-year overall survival rate observed in this study (76.4%) aligns with a previous report of the 30-year overall survival rate in 256 patients with APDS (74%), among whom no significant difference in overall survival between APDS1 and APDS2 was also observed [17]. To our knowledge, our study provides the most current and comprehensive estimate of overall survival among patients with APDS [12, 16, 17].

In this study, a divergence in survival between patients with APDS and the global population was observed beginning in adolescence and sustained through adulthood. Nearly all patients with APDS (98%) experience their first symptoms of APDS in infancy or childhood at a median age of 2.0 years (range, birth to 22 years). In contrast, the median age of diagnosis is 13.4 years (range, 0–56 years) [18]. Delays in APDS diagnosis of a median 7.0 years (IQR, 3.4–14.0 years) and mean 10.6 years (range, 0–44 years) have been previously reported [18, 122]. The mortality implications of early onset of APDS manifestations coupled with delays in diagnosis and lack of effective early interventions are not fully understood. However, findings from our study suggest that timely diagnosis and effective management of APDS at first presentation of symptoms may improve survival in these patients but should be confirmed in future analyses of OS relative to the diagnostic timing.

Our findings also highlight the exacerbated mortality of patients with APDS and concurrent lymphoma, with a median overall survival of 41 years. The cumulative risk of lymphoid malignancy has been previously estimated to be as high as 78% at 40 years of age, and up to 62% of APDS fatalities may be attributable to lymphoma [12, 123]. In this study, the overall survival rate in patients with APDS and concurrent lymphoma was 42.7% at 41 years of age, compared with 66.8% in the overall APDS population at the same age. Although we did not observe a significant difference in overall survival between patients with APDS with and without concurrent lymphoma, an empirical assessment of the data suggests an accelerated decline in survival among patients with APDS and concurrent lymphoma relative to those without concurrent lymphoma and to the overall APDS population. Thus, our findings underscore the considerable influence of concurrent lymphoma on overall survival and emphasize the importance of focusing mitigation efforts on decreasing the incidence of lymphoma in patients with APDS.

Previous literature suggests that HSCT can reverse some phenotypes of APDS or achieve a cure; the reported overall survival rates after HSCT were 81% over follow-up periods ranging from 8 months to 16 years and 86% at 2 years [14, 15]. However, prior studies have also reported high rates of complications following HSCT, including graft instability or failure and severe infection [14–16]. In this study, we observed that the estimated overall survival of patients with APDS remained largely unchanged when censoring for HSCT. Moreover, no significant difference was noted when overall survival in patients with APDS who were reported to have undergone HSCT was compared with overall survival in those who had not. With the data available at the time of this analysis, our results suggest that HSCT may not provide a meaningful clinical benefit in survival in patients with APDS and ongoing evaluation is warranted. Further investigations are warranted to assess whether the benefits observed with HSCT outweigh the risk of adverse complications including graft failure or instability, poor graft function, graft vs. host disease, and mortality [14–17, 122].

The results of this study should be considered within the context of its limitations. First, this study relied on published data, potentially introducing publication bias. However, this study includes a large collection of publications describing patients with APDS across multiple countries to help mitigate bias. Secondly, age-related information in the literature predominantly focuses on early decades of life, resulting in fewer data available for the construction of Kaplan-Meier curves for later decades. Given the recent characterization of APDS in 2013 and a median age of diagnosis of 12 years, the distribution of age among patients with APDS observed in our study may be reflective of real-world trends [1, 2, 122]. Additionally, the follow-up time after HSCT was based on follow-up times noted in the literature. Therefore, the time between age at HSCT and last age observed may not have been sufficient to capture the long-term survival benefit associated with transplant. Likewise, literature references reported that only 13 patients received leniolisib, which precluded the assessment of the impact of leniolisib on overall survival. With the approval of leniolisib for treatment of APDS in 2023 by the US Food and Drug Administration, future studies may extend the present findings by evaluating its impact on mortality. Additionally, the creation of an *International Classification of Diseases*,* Tenth Revision*,* Clinical Modification* (*ICD-10-CM*) code for APDS (D81.82) in 2023 may also help identify patients for future analyses. Finally, due to lack of individual data for the global population, CIs for survival estimates could not be calculated in this population, and a statistical comparison of overall survival between the overall APDS population and global population was not feasible. Despite these limitations, our assessment of APDS mortality relative to the global population sheds light on the considerable burden of this disease. Moreover, our study assesses the largest number of patients with APDS for whom survival has been evaluated, increasing the generalizability of our findings to the broader APDS population, and it is the first study to evaluate the impact of concurrent lymphoma and HSCT on survival.

## Conclusions

This study provides the most current and comprehensive estimate of overall survival in patients with APDS. Relative to the global population, the overall survival rate was lower among the overall APDS population and across APDS subtype, with no difference in mortality between APDS1 and APDS2. The observed lack of improvement in survival after HSCT warrants further investigation of the impact of this therapy in patients with APDS. Findings from this study indicate a high disease burden associated with APDS, particularly in patients with concurrent lymphoma, highlighting the unmet need for disease-modifying treatments to improve survival in this patient population.

## Data Availability

Data used within this manuscript are available in PubMed and Embase.

## References

[1] Angulo I, Vadas O, Garçon F, Banham-Hall E, Plagnol V, Leahy TR, et al. Phosphoinositide 3-kinase δ gene mutation predisposes to respiratory infection and airway damage. Science. 2013;342(6160):866–71. 24136356 10.1126/science.1243292 PMC3930011

[2] Lucas CL, Zhang Y, Venida A, Wang Y, Hughes J, McElwee J, et al. Heterozygous splice mutation in PIK3R1 causes human immunodeficiency with lymphoproliferation due to dominant activation of PI3K. J Exp Med. 2014;211(13):2537–47. 25488983 10.1084/jem.20141759 PMC4267241

[3] Vanselow S, Wahn V, Schuetz C. Activated PI3Kδ syndrome - reviewing challenges in diagnosis and treatment. Front Immunol. 2023;14:1208567. 37600808 10.3389/fimmu.2023.1208567 PMC10432830

[4] Coulter TI, Cant AJ. The treatment of activated PI3Kδ syndrome. Front Immunol. 2018;9:2043. 30245694 10.3389/fimmu.2018.02043 PMC6137162

[5] Michalovich D, Nejentsev S. Activated PI3 kinase delta syndrome: from genetics to therapy. Front Immunol. 2018;9:369. 29535736 10.3389/fimmu.2018.00369 PMC5835040

[6] Oh J, Garabedian E, Fuleihan R, Cunningham-Rundles C. Clinical manifestations and outcomes of activated phosphoinositide 3-kinase δ syndrome from the USIDNET cohort. J Allergy Clin Immunol Pract. 2021;9(11):4095–102. 34352450 10.1016/j.jaip.2021.07.044 PMC8578310

[7] Lucas CL, Kuehn HS, Zhao F, Niemela JE, Deenick EK, Palendira U, et al. Dominant-activating germline mutations in the gene encoding the PI(3)K catalytic subunit p110δ result in T cell senescence and human immunodeficiency. Nat Immunol. 2014;15(1):88–97. 24165795 10.1038/ni.2771 PMC4209962

[8] Deau MC, Heurtier L, Frange P, Suarez F, Bole-Feysot C, Nitschke P, et al. A human immunodeficiency caused by mutations in the PIK3R1 gene. J Clin Invest. 2014;124(9):3923–8. 25133428 10.1172/JCI75746 PMC4153704

[9] Cant AJ, Chandra A, Munro E, Rao VK, Lucas CL. PI3Kδ pathway dysregulation and unique features of its inhibition by leniolisib in activated PI3Kδ syndrome and beyond. J Allergy Clin Immunol Pract. 2024;12(1):69–78. 37777067 10.1016/j.jaip.2023.09.016 PMC10872751

[10] Nunes-Santos CJ, Uzel G, Rosenzweig SD. PI3K pathway defects leading to immunodeficiency and immune dysregulation. J Allergy Clin Immunol. 2019;143(5):1676–87. 31060715 10.1016/j.jaci.2019.03.017

[11] Coulter TI, Chandra A, Bacon CM, Babar J, Curtis J, Screaton N, et al. Clinical spectrum and features of activated phosphoinositide 3-kinase δ syndrome: a large patient cohort study. J Allergy Clin Immunol. 2017;139(2):597–606.e4. 27555459 10.1016/j.jaci.2016.06.021 PMC5292996

[12] Elkaim E, Neven B, Bruneau J, Mitsui-Sekinaka K, Stanislas A, Heurtier L, et al. Clinical and immunologic phenotype associated with activated phosphoinositide 3-kinase δ syndrome 2: a cohort study. J Allergy Clin Immunol. 2016;138(1):210–8.e9. 27221134 10.1016/j.jaci.2016.03.022

[13] Maccari ME, Abolhassani H, Aghamohammadi A, Aiuti A, Aleinikova O, Bangs C, et al. Disease evolution and response to rapamycin in activated phosphoinositide 3-kinase δ syndrome: the European Society for Immunodeficiencies-Activated Phosphoinositide 3-Kinase δ Syndrome Registry. Front Immunol. 2018;9:543. 29599784 10.3389/fimmu.2018.00543 PMC5863269

[14] Dimitrova D, Nademi Z, Maccari ME, Ehl S, Uzel G, Tomoda T, et al. International retrospective study of allogeneic hematopoietic cell transplantation for activated PI3K-delta syndrome. J Allergy Clin Immunol. 2022;149(1):410–21.e7. 34033842 10.1016/j.jaci.2021.04.036 PMC8611111

[15] Nademi Z, Slatter MA, Dvorak CC, Neven B, Fischer A, Suarez F, et al. Hematopoietic stem cell transplant in patients with activated PI3K delta syndrome. J Allergy Clin Immunol. 2017;139(3):1046–9. 27847301 10.1016/j.jaci.2016.09.040

[16] Okano T, Imai K, Tsujita Y, Mitsuiki N, Yoshida K, Kamae C, et al. Hematopoietic stem cell transplantation for progressive combined immunodeficiency and lymphoproliferation in patients with activated phosphatidylinositol-3-OH kinase δ syndrome type 1. J Allergy Clin Immunol. 2019;143(1):266–75. 29778502 10.1016/j.jaci.2018.04.032

[17] Hanson J, Bonnen PE. Systematic review of mortality and survival rates for APDS. Clin Exp Med. 2024;24(1):17. 38280023 10.1007/s10238-023-01259-y PMC10821986

[18] Büsch K, Memmott HL, McLaughlin HM, Upton JEM, Harrington A. Genetic etiologies and outcomes in malignancy and mortality in activated phosphoinositide 3-kinase delta syndrome: a systematic review. Adv Ther.2025;42(2):752–771. 39636570 10.1007/s12325-024-03066-7 PMC11787279

[19] Methley AM, Campbell S, Chew-Graham C, McNally R, Cheraghi-Sohi S. PICO, PICOS and SPIDER: a comparison study of specificity and sensitivity in three search tools for qualitative systematic reviews. BMC Health Serv Res. 2014;14:579. 25413154 10.1186/s12913-014-0579-0 PMC4310146

[20] Page MJ, McKenzie JE, Bossuyt PM, Boutron I, Hoffmann TC, Mulrow CD, et al. The PRISMA 2020 statement: an updated guideline for reporting systematic reviews. BMJ. 2021;372:n71. 33782057 10.1136/bmj.n71 PMC8005924

[21] Zhou Z, Zondag T, Hermans M, van Hagen PM, van Laar JAM. Hemophagocytic lymphohistiocytosis in activated PI3K delta syndrome: an illustrative case report. J Clin Immunol. 2021;41(7):1656–9. 34115277 10.1007/s10875-021-01080-w PMC8193594

[22] Conti F, Catelli A, Cifaldi C, Leonardi L, Mulè R, Fusconi M, et al. Case report: Hodgkin lymphoma and refractory systemic lupus erythematosus unveil activated phosphoinositide 3-kinase-δ syndrome 2 in an adult patient. Front Pediatr. 2021;9:702546. 34307262 10.3389/fped.2021.702546 PMC8295470

[23] Yin J, Ma J, Xia J, Cao Y, Li C. Activated PI3Kδ syndrome 1 mimicking systemic lupus erythematosus and secondary Sjögren’s syndrome-like phenotype without recurrent infections: a case report. Front Pediatr. 2022;10:1077324. 36605759 10.3389/fped.2022.1077324 PMC9807900

[24] Ahmed AA, El Shahaway AA, Hussien SA. Activated PI3K-delta syndrome in an Egyptian pediatric cohort with primary immune deficiency. Allergol Immunopathol (Madr). 2020;48(6):686–93. 32349894 10.1016/j.aller.2019.12.006

[25] Baleydier F, Ranza E, Schäppi M, Rougemont AL, Merlini L, Ansari M, et al. Activated phosphoinositide 3 kinase delta syndrome (APDS): a primary immunodeficiency mimicking lymphoma. J Pediatr Hematol Oncol. 2019;41(8):e521–4. 30334905 10.1097/MPH.0000000000001328

[26] Bloomfield M, Klocperk A, Zachova R, Milota T, Kanderova V, Sediva A. Natural course of activated phosphoinositide 3-kinase delta syndrome in childhood and adolescence. Front Pediatr. 2021;9:697706. 34350147 10.3389/fped.2021.697706 PMC8326455

[27] Bravo García-Morato M, García-Miñaúr S, Molina Garicano J, Santos Simarro F, Del Pino Molina L, López-Granados E, et al. Mutations in PIK3R1 can lead to APDS2, SHORT syndrome or a combination of the two. Clin Immunol. 2017;179:77–80. 28302518 10.1016/j.clim.2017.03.004

[28] Buchbinder D, Seppanen M, Rao VK, Uzel G, Nugent D. Clinical challenges: identification of patients with novel primary immunodeficiency syndromes. J Pediatr Hematol Oncol. 2018;40(5):e319–22. 29200144 10.1097/MPH.0000000000001003 PMC5984070

[29] Cansever M, Zietara N, Chiang SCC, Ozcan A, Yilmaz E, Karakukcu M, et al. A rare case of activated phosphoinositide 3-kinase delta syndrome (APDS) presenting with hemophagocytosis complicated with Hodgkin lymphoma. J Pediatr Hematol Oncol. 2020;42(2):156–9. 31033788 10.1097/MPH.0000000000001487

[30] Ceraulo A, Malcus C, Durandy A, Picard C, Bertrand Y. Activated PI3-kinase δ syndrome: long-term follow-up after cord blood transplantation. J Clin Immunol. 2016;36(6):544–6. 27294376 10.1007/s10875-016-0305-2

[31] Chiriaco M, Brigida I, Ariganello P, Di Cesare S, Di Matteo G, Taus F, et al. The case of an APDS patient: defects in maturation and function and decreased in vitro anti-mycobacterial activity in the myeloid compartment. Clin Immunol. 2017;178:20–8. 26732860 10.1016/j.clim.2015.12.008

[32] Craig M, Geng B, Wigby K, Phillips SA, Bakhoum C, Naheedy J, et al. Activated phosphoinositide 3-kinase δ syndrome associated with nephromegaly, growth hormone deficiency, bronchiectasis: a case report. Allergy Asthma Clin Immunol. 2022;18(1):15. 35189965 10.1186/s13223-022-00655-5 PMC8862239

[33] Crank MC, Grossman JK, Moir S, Pittaluga S, Buckner CM, Kardava L, et al. Mutations in PIK3CD can cause hyper IgM syndrome (HIGM) associated with increased cancer susceptibility. J Clin Immunol. 2014;34(3):272–6. 24610295 10.1007/s10875-014-0012-9 PMC4159085

[34] Diaz N, Juarez M, Cancrini C, Heeg M, Soler-Palacín P, Payne A, et al. Seletalisib for activated PI3Kδ syndromes: open-label phase 1b and extension studies. J Immunol. 2020;205(11):2979–87. 33115853 10.4049/jimmunol.2000326

[35] Dominguez-Pinilla N, Allende LM, Rosain J, Gallego MDC, Chaves F, Deswarte C, et al. Disseminated abscesses due to Mycoplasma faucium in a patient with activated PI3Kδ syndrome type 2. J Allergy Clin Immunol Pract. 2018;6(5):1796–8.e2. 29486251 10.1016/j.jaip.2018.02.014

[36] Donaldson SL, Purnell JC, Pavlidakey PG, Atkinson TP, Kissel R. Epidermodysplasia verruciformis in a young adult with activated PI3Kδ syndrome. JAAD Case Rep. 2019;5(2):195–7. 30740505 10.1016/j.jdcr.2018.10.026 PMC6357544

[37] Dulau Florea AE, Braylan RC, Schafernak KT, Williams KW, Daub J, Goyal RK, et al. Abnormal B-cell maturation in the bone marrow of patients with germline mutations in PIK3CD. J Allergy Clin Immunol. 2017;139(3):1032–5.e6. 27697496 10.1016/j.jaci.2016.08.028 PMC5342918

[38] Edwards ESJ, Bier J, Cole TS, Wong M, Hsu P, Berglund LJ, et al. Activating PIK3CD mutations impair human cytotoxic lymphocyte differentiation and function and EBV immunity. J Allergy Clin Immunol. 2019;143(1):276–91.e6. 29800648 10.1016/j.jaci.2018.04.030

[39] Elgizouli M, Lowe DM, Speckmann C, Schubert D, Hülsdünker J, Eskandarian Z, et al. Activating PI3Kδ mutations in a cohort of 669 patients with primary immunodeficiency. Clin Exp Immunol. 2016;183(2):221–9. 26437962 10.1111/cei.12706 PMC4711166

[40] Ewertowska M, Grześk E, Urbańczyk A, Dąbrowska A, Bąbol-Pokora K, Łęcka M, et al. Activated phosphoinositide 3-kinase delta syndrome 1 and 2 (APDS 1 and APDS 2): similarities and differences based on clinical presentation in two boys. Allergy Asthma Clin Immunol. 2020;16:22. 32265996 10.1186/s13223-020-00420-6 PMC7115069

[41] Fang S, Zeng A, Xu Q, Zhou L, Zhang Z, An Y, et al. Generation of human induced pluripotent stem cell line from peripheral blood mononuclear cells from an activated phosphoinositide 3-kinase δ syndrome patient. Stem Cell Res. 2022;62:102822. 35660815 10.1016/j.scr.2022.102822

[42] Fox TA, Chakraverty R, Burns S, Carpenter B, Thomson K, Lowe D, et al. Successful outcome following allogeneic hematopoietic stem cell transplantation in adults with primary immunodeficiency. Blood. 2018;131(8):917–31. 29279357 10.1182/blood-2017-09-807487 PMC6225386

[43] Fuentes LA, Garkaby J, Scott O, Pachul JW, Dadi H, Vong L. Novel mutation in *PIK3CD* affecting the Ras-binding domain. LymphoSign J. 2022;9(1):11–6.

[44] Hartman HN, Niemela J, Hintermeyer MK, Garofalo M, Stoddard J, Verbsky JW, et al. Gain of function mutations of PIK3CD as a cause of primary sclerosing cholangitis. J Clin Immunol. 2015;35(1):11–4. 25352054 10.1007/s10875-014-0109-1 PMC4769101

[45] Hauck F, Magg T, Krolo A, Bilic I, Hirschmugl T, Laass M, et al. Variant PIK3R1 hypermorphic mutation and clinical phenotypes in a family with short statures, mild immunodeficiency and lymphoma. Klin Padiatr. 2017;229(3):113–7. 28561224 10.1055/s-0043-104218

[46] Heurtier L, Lamrini H, Chentout L, Deau MC, Bouafia A, Rosain J, et al. Mutations in the adaptor-binding domain and associated linker region of p110δ cause activated PI3K-δ syndrome 1 (APDS1). Haematologica. 2017;102(7):e278–81. 28428270 10.3324/haematol.2017.167601 PMC5566055

[47] Hong Y, Nanthapisal S, Omoyinmi E, Olbrich P, Neth O, Speckmann C, et al. Secondary C1q deficiency in activated PI3Kδ syndrome type 2. Front Immunol. 2019;10:2589. 31781101 10.3389/fimmu.2019.02589 PMC6859795

[48] Hong CR, Lee S, Hong KT, Choi JY, Shin HY, Choi M, et al. Successful haploidentical transplantation with post-transplant cyclophosphamide for activated phosphoinositide 3-kinase δ syndrome. J Allergy Clin Immunol Pract. 2019;7(3):1034–7.e1. 29890288 10.1016/j.jaip.2018.05.029

[49] Inglés-Ferrándiz M, Martin-Inaraja M, Herrera L, Villaverde M, Santos S, Vesga MA, et al. Generation, establishment and characterization of a pluripotent stem cell line (CVTTHi001-A) from primary fibroblasts isolated from a patient with activated PI3 kinase delta syndrome (APDS2). Stem Cell Res. 2020;49:102082. 33221676 10.1016/j.scr.2020.102082

[50] Kang JM, Kim SK, Kim D, Choi SR, Lim YJ, Kim SK, et al. Successful sirolimus treatment for Korean patients with activated phosphoinositide 3-kinase δ syndrome 1: the first case series in Korea. Yonsei Med J. 2020;61(6):542–6. 32469178 10.3349/ymj.2020.61.6.542 PMC7256007

[51] Kannan JA, Dávila-Saldaña BJ, Zhang K, Filipovich AH, Kucuk ZY. Activated phosphoinositide 3-kinase δ syndrome in a patient with a former diagnosis of common variable immune deficiency, bronchiectasis, and lymphoproliferative disease. Ann Allergy Asthma Immunol. 2015;115(5):452–4. 26371693 10.1016/j.anai.2015.08.009

[52] Karanovic D, Michelow IC, Hayward AR, DeRavin SS, Delmonte OM, Grigg ME, et al. Disseminated and congenital toxoplasmosis in a mother and child with activated PI3-kinase δ syndrome type 2 (APDS2): case report and a literature review of toxoplasma infections in primary immunodeficiencies. Front Immunol. 2019;10:77. 30891027 10.3389/fimmu.2019.00077 PMC6413717

[53] Kracker S, Curtis J, Ibrahim MA, Sediva A, Salisbury J, Campr V, et al. Occurrence of B-cell lymphomas in patients with activated phosphoinositide 3-kinase δ syndrome. J Allergy Clin Immunol. 2014;134(1):233–6. 24698326 10.1016/j.jaci.2014.02.020 PMC4671279

[54] Kralickova P, Milota T, Litzman J, Malkusova I, Jilek D, Petanova J, et al. CVID-associated tumors: Czech nationwide study focused on epidemiology, immunology, and genetic background in a cohort of patients with CVID. Front Immunol. 2018;9:3135. 30723478 10.3389/fimmu.2018.03135 PMC6349737

[55] Kuhlen M, Hönscheid A, Loizou L, Nabhani S, Fischer U, Stepensky P, et al. De Novo PIK3R1 gain-of-function with recurrent sinopulmonary infections, long-lasting chronic CMV-lymphadenitis and microcephaly. Clin Immunol. 2016;162:27–30. 26529633 10.1016/j.clim.2015.10.008

[56] Larrauffie A, Syrykh C, Tavitian S, Comont T, Dion J. Activated PI3 kinase delta syndrome revealed by vasculitis and disseminated toxoplasmosis. J Clin Immunol. 2022;42(3):688–90. 35022947 10.1007/s10875-021-01186-1

[57] Lawrence MG, Uzel G. 6-year-old boy with recurrent sinopulmonary infections and lymphadenopathy. J Allergy Clin Immunol Pract. 2015;3(3):461–3.e1. quiz 4–5. 25956317 10.1016/j.jaip.2014.10.017

[58] Li GM, Liu HM, Guan WZ, Xu H, Wu BB, Feng JY, et al. A mutation in PIK3CD gene causing pediatric systemic lupus erythematosus: a case report. Med (Baltim). 2019;98(18):e15329. 10.1097/MD.0000000000015329 PMC6504300 31045771

[59] Lougaris V, Faletra F, Lanzi G, Vozzi D, Marcuzzi A, Valencic E, et al. Altered germinal center reaction and abnormal B cell peripheral maturation in PI3KR1-mutated patients presenting with HIGM-like phenotype. Clin Immunol. 2015;159(1):33–6. 25939554 10.1016/j.clim.2015.04.014

[60] Lu M, Gu W, Sheng Y, Wang J, Xu X. Case report: activating PIK3CD mutation in patients presenting with granulomatosis with polyangiitis. Front Immunol. 2021;12:670312. 33995405 10.3389/fimmu.2021.670312 PMC8113859

[61] Lugo Reyes SO, Solórzano Suárez A, Scheffler Mendoza SC, Xóchihua Díaz L, González Serrano ME, López Herrera G, et al. Activating de novo monoallelic variants causing inborn errors of immunity in two unrelated children born of HIV-seroconcordant couples. Aids. 2022;36(15):2121–8. 36382434 10.1097/QAD.0000000000003367

[62] Luo Y, Xia Y, Wang W, Li Z, Jin Y, Gong Y, et al. Identification of a novel de novo gain-of-function mutation of PIK3CD in a patient with activated phosphoinositide 3-kinase δ syndrome. Clin Immunol. 2018;197:60–7. 30138677 10.1016/j.clim.2018.08.007

[63] Maffucci P, Filion CA, Boisson B, Itan Y, Shang L, Casanova JL, et al. Genetic diagnosis using whole exome sequencing in common variable immunodeficiency. Front Immunol. 2016;7:220. 27379089 10.3389/fimmu.2016.00220 PMC4903998

[64] Mandola AB, Dadi H, Reid B, Roifman CM. Novel heterozygous PIK3CD mutation presenting with only laboratory markers of combined immunodeficiency. LymphoSign J. 2020;7(2):49–55.

[65] Martínez-Saavedra MT, García-Gomez S, Domínguez Acosta A, Mendoza Quintana JJ, Páez JP, García-Reino EJ, et al. Gain-of-function mutation in PIK3R1 in a patient with a narrow clinical phenotype of respiratory infections. Clin Immunol. 2016;173:117–20. 27693481 10.1016/j.clim.2016.09.011

[66] Marzollo A, Bresolin S, Colavito D, Cani A, Gaio P, Bosa L, et al. Case report: intestinal nodular lymphoid hyperplasia as first manifestation of activated PI3Kδ syndrome due to a novel PIK3CD variant. Front Pediatr. 2021;9:703056. 34692603 10.3389/fped.2021.703056 PMC8528001

[67] Mettman D, Thiffault I, Dinakar C, Saunders C. Immunodeficiency-associated lymphoid hyperplasia as a cause of intussusception in a case of activated PI3K-δ syndrome. Front Pediatr. 2017;5:71. 28469999 10.3389/fped.2017.00071 PMC5395656

[68] Moreno-Corona N, Chentout L, Poggi L, Thouenon R, Masson C, Parisot M, et al. Two monogenetic disorders, activated PI3-kinase-δ syndrome 2 and Smith-Magenis syndrome, in one patient: case report and a literature review of neurodevelopmental impact in primary immunodeficiencies associated with disturbed PI3K signaling. Front Pediatr. 2021;9:688022. 34249818 10.3389/fped.2021.688022 PMC8266209

[69] Nakagawa R, Takasawa K, Yeh TW, Imai K, Kashimada K, Morio T. Type 1 diabetes mellitus associated with activated phosphatidylinositol 3-kinase delta syndrome, type 2. J Diabetes. 2018;10(5):421–2. 29280567 10.1111/1753-0407.12638

[70] Nguyen Y, Rosain J, Aguilar C, Picard C, Malphettes M. Long-term follow-up of an activated PI3K-δ syndrome 2 in patient presenting with an agammaglobulinemia phenotype. Ann Allergy Asthma Immunol. 2018;121(6):739–40.e1. 30081089 10.1016/j.anai.2018.07.043

[71] Orf K, Abbas A, Abdel-Aziz K, Burns SO. Transverse myelitis in a patient with activated phosphoinositide 3-kinase δ syndrome type 1. Clin Immunol. 2020;219:108552. 32758532 10.1016/j.clim.2020.108552

[72] Petrovski S, Parrott RE, Roberts JL, Huang H, Yang J, Gorentla B, et al. Dominant splice site mutations in PIK3R1 cause hyper IgM syndrome, lymphadenopathy and short stature. J Clin Immunol. 2016;36(5):462–71. 27076228 10.1007/s10875-016-0281-6 PMC5690581

[73] Pham MN, Cunningham-Rundles C. Evaluation of lymphoproliferative disease and increased risk of lymphoma in activated phosphoinositide 3 kinase delta syndrome: a case report with discussion. Front Pediatr. 2018;6:402. 30619796 10.3389/fped.2018.00402 PMC6305443

[74] Qiu L, Wang Y, Tang W, Yang Q, Zeng T, Chen J, et al. Activated phosphoinositide 3-kinase δ syndrome: a large pediatric cohort from a single center in China. J Clin Immunol. 2022;42(4):837–50. 35296988 10.1007/s10875-022-01218-4

[75] Wang Y, Yang Q, Chen X, Tang W, Zhou L, Chen Z, et al. Phenotypic characterization of patients with activated PI3Kδ syndrome 1 presenting with features of systemic lupus erythematosus. Genes Dis. 2021;8(6):907–17. 34522717 10.1016/j.gendis.2020.04.012 PMC8427252

[76] Rae W, Gao Y, Ward D, Mattocks CJ, Eren E, Williams AP. A novel germline gain-of-function variant in PIK3CD. Clin Immunol. 2017;181:29–31. 28578023 10.1016/j.clim.2017.05.020

[77] Rae W, Ramakrishnan KA, Gao Y, Ashton-Key M, Pengelly RJ, Patel SV, et al. Precision treatment with sirolimus in a case of activated phosphoinositide 3-kinase δ syndrome. Clin Immunol. 2016;171:38–40. 27444043 10.1016/j.clim.2016.07.017

[78] Ramirez L, Tamayo W, Ale H. APDS2 and SHORT syndrome in a teenager with PIK3R1 pathogenic variant. J Clin Immunol. 2020;40(7):1020–5. 32778990 10.1007/s10875-020-00843-1

[79] Rao VK, Webster S, Dalm V, Šedivá A, van Hagen PM, Holland S, et al. Effective “activated PI3Kδ syndrome”-targeted therapy with the PI3Kδ inhibitor leniolisib. Blood. 2017;130(21):2307–16. 28972011 10.1182/blood-2017-08-801191 PMC5701526

[80] Rivalta B, Amodio D, Milito C, Chiriaco M, Di Cesare S, Giancotta C, et al. Case report: EBV chronic infection and lymphoproliferation in four APDS patients: the challenge of proper characterization, therapy, and follow-up. Front Pediatr. 2021;9:703853. 34540765 10.3389/fped.2021.703853 PMC8448282

[81] Rowane MJ, Callahan MA, Schend JE, Rowane MP, Hostoffer RW. Structural abnormalities and osteopathic considerations in primary immunodeficiencies. J Osteopath Med. 2023;123(4):195–9. 36692027 10.1515/jom-2022-0129

[82] Ruiz-García R, Vargas-Hernández A, Chinn IK, Angelo LS, Cao TN, Coban-Akdemir Z, et al. Mutations in PI3K110δ cause impaired natural killer cell function partially rescued by rapamycin treatment. J Allergy Clin Immunol. 2018;142(2):605–17.e7. 29330011 10.1016/j.jaci.2017.11.042 PMC6109967

[83] Sanchez Clemente N, Penner J, Breuer J, Ip W, Booth C. Case report: a severe paediatric presentation of COVID-19 in APDS2 immunodeficiency. Front Immunol. 2022;13:881259. 35707532 10.3389/fimmu.2022.881259 PMC9190774

[84] Saunders JL, O’Connor MG, Machogu EM. Nasal nitric oxide may not differentiate primary ciliary dyskinesia from certain primary immunodeficiencies. Pediatr Pulmonol. 2022;57(9):2269–72. 35596239 10.1002/ppul.25989

[85] Schworer SA, Francis OL, Johnson SM, Smith BD, Gold SH, Smitherman AB, et al. Autoimmune cytopenia as an early and initial presenting manifestation in activated PI3 kinase delta syndrome: case report and review. J Pediatr Hematol Oncol. 2021;43(8):281–7. 34054047 10.1097/MPH.0000000000002214 PMC8542580

[86] Segundo GRS, Takano OA, Moraes LSL, Nadaf M, Fernandes SJ, Ochs HD, et al. Paternal gonadal mosaicism as cause of a puzzling inheritance pattern of activated PI3-kinase delta syndrome. Ann Allergy Asthma Immunol. 2017;119(6):564–6. 29107464 10.1016/j.anai.2017.09.054

[87] Stray-Pedersen A, Sorte HS, Samarakoon P, Gambin T, Chinn IK, Coban Akdemir ZH, et al. Primary immunodeficiency diseases: genomic approaches delineate heterogeneous Mendelian disorders. J Allergy Clin Immunol. 2017;139(1):232–45. 27577878 10.1016/j.jaci.2016.05.042 PMC5222743

[88] Su G, Lai J, Zhu J, Zhang D, Hou J, Xu Y, et al. Analysis of five cases of monogenic lupus related to primary immunodeficiency diseases. Inflamm Res. 2021;70(10–12):1211–6. 34559261 10.1007/s00011-021-01479-6

[89] Sugiyama M, Iguchi A, Yamada M, Terashita Y, Ohshima J, Cho Y, et al. Successful bone marrow transplantation in two sisters with activated phosphoinositide 3-kinase δ syndrome 2. Bone Marrow Transpl. 2017;52(12):1678–80. 10.1038/bmt.2017.189 28869616

[90] Sun B, Zhou S, Yang H, Zhou J, Leng X, Zhang W, et al. Tofacitinib as a possible treatment for arthritis in an APDS2 patient. Rheumatology (Oxford). 2023;62(3):e39–41. 35904543 10.1093/rheumatology/keac436

[91] Szczawińska-Popłonyk A, Bernat-Sitarz K, Schwartzmann E, Piechota M, Badura-Stronka M. Clinical and immunological assessment of APDS2 with features of the SHORT syndrome related to a novel mutation in PIK3R1 with reduced penetrance. Allergol Immunopathol (Madr). 2022;50(4):1–9. 35789397 10.15586/aei.v50i4.510

[92] Takeda AJ, Zhang Y, Dornan GL, Siempelkamp BD, Jenkins ML, Matthews HF, et al. Novel PIK3CD mutations affecting N-terminal residues of p110δ cause activated PI3Kδ syndrome (APDS) in humans. J Allergy Clin Immunol. 2017;140(4):1152–6.e10. 28414062 10.1016/j.jaci.2017.03.026 PMC5632585

[93] Tessarin G, Rossi S, Baronio M, Gazzurelli L, Colpani M, Benvenuto A et al. Activated phosphoinositide 3-kinase delta syndrome 1: clinical and immunological data from an Italian cohort of patients. J Clin Med. 2020;9(10). 10.3390/jcm9103335 PMC7603210 33080915

[94] Thauland TJ, Pellerin L, Ohgami RS, Bacchetta R, Butte MJ. Case study: mechanism for increased follicular helper T cell development in activated PI3K delta syndrome. Front Immunol. 2019;10:753. 31031754 10.3389/fimmu.2019.00753 PMC6473200

[95] Valencic E, Grasso AG, Conversano E, Lucafò M, Piscianz E, Gregori M, et al. Theophylline as a precision therapy in a young girl with PIK3R1 immunodeficiency. J Allergy Clin Immunol Pract. 2018;6(6):2165–7. 29510232 10.1016/j.jaip.2018.02.029

[96] Wallace JG, Zambrano-Rodas P, Córdova-Calderón W, Estrada-Turriate S, Mendoza-Quispe D, Limache Ontiveros Y, et al. Dysregulated actin dynamics in activated PI3Kδ syndrome. Clin Immunol. 2020;210:108311. 31760094 10.1016/j.clim.2019.108311 PMC6989370

[97] Wang W, Min Q, Lai N, Csomos K, Wang Y, Liu L, et al. Cellular mechanisms underlying B cell abnormalities in patients with gain-of-function mutations in the PIK3CD gene. Front Immunol. 2022;13:890073. 35799777 10.3389/fimmu.2022.890073 PMC9253290

[98] Wentink M, Peeters D, van der Burg M, Vermont C, Duijts L, Driessen GJ. A 3-year-old girl with a mediastinal mass. Chest. 2019;155(1):e13–6. 30616742 10.1016/j.chest.2018.07.042

[99] Wentink M, Dalm V, Lankester AC, van Schouwenburg PA, Schölvinck L, Kalina T, et al. Genetic defects in PI3Kδ affect B-cell differentiation and maturation leading to hypogammaglobulineamia and recurrent infections. Clin Immunol. 2017;176:77–86. 28104464 10.1016/j.clim.2017.01.004

[100] Williams SN. Endoscopic airway manifestations in a pediatric patient with activated PI3K-delta syndrome. Pediatr Pulmonol. 2020;55(11):2836–7. 32969594 10.1002/ppul.25021

[101] Yang X, Xi R, Bai J, Pan Y. Successful haploidentical hematopoietic stem cell transplantation for activated phosphoinositide 3-kinase δ syndrome: case report and literature review. Med (Baltim). 2023;102(5):e32816. 10.1097/MD.0000000000032816 PMC9902017 36749229

[102] Yazdani R, Hamidi Z, Babaha F, Azizi G, Fekrvand S, Abolhassani H, et al. PIK3R1 mutation associated with hyper IgM (APDS2 syndrome): a case report and review of the literature. Endocr Metab Immune Disord Drug Targets. 2019;19(7):941–58. 30799802 10.2174/1871530319666190225114739

[103] Yin Z, Tian X, Zou R, He X, Chen K, Zhu C. Case report: first occurrence of plasmablastic lymphoma in activated phosphoinositide 3-kinase δ syndrome. Front Immunol. 2021;12:813261. 34992612 10.3389/fimmu.2021.813261 PMC8724197

[104] Zhang X, Wang J, Zhu K, Jin Y, Fu H, Mao J. Activated phosphoinositide 3-kinase delta syndrome misdiagnosed as anti-neutrophil cytoplasmic antibody-associated vasculitis: a case report. J Int Med Res. 2021;49(5):3000605211013222. 34039074 10.1177/03000605211013222 PMC8755648

[105] Zhang Q, Ma H, Ma J, Wang D, Zhao Y, Wang T, et al. Clinical and genetic analysis of immunodeficiency-related diseases associated with PIK3CD mutations. Pediatr Investig. 2018;2(4):257–62. 32851276 10.1002/ped4.12101 PMC7331349

[106] Ye X, Maglione PJ, Wehr C, Li X, Wang Y, Abolhassani H, et al. Genomic characterization of lymphomas in patients with inborn errors of immunity. Blood Adv. 2022;6(18):5403–14. 35687490 10.1182/bloodadvances.2021006654 PMC9631701

[107] Asano T, Okada S, Tsumura M, Yeh TW, Mitsui-Sekinaka K, Tsujita Y, et al. Enhanced AKT phosphorylation of circulating B cells in patients with activated PI3Kδ syndrome. Front Immunol. 2018;9:568. 29675019 10.3389/fimmu.2018.00568 PMC5895775

[108] Inoue M, Isoda T, Yamashita M, Tomoda T, Inoue K, Okano T, et al. Cytomegalovirus laryngitis in primary combined immunodeficiency diseases. J Clin Immunol. 2021;41(1):243–7. 33033934 10.1007/s10875-020-00873-9

[109] Olbrich P, Lorenz M, Cura Daball P, Lucena JM, Rensing-Ehl A, Sanchez B, et al. Activated PI3Kδ syndrome type 2: two patients, a novel mutation, and review of the literature. Pediatr Allergy Immunol. 2016;27(6):640–4. 27116393 10.1111/pai.12585

[110] Goto F, Uchiyama T, Nakazawa Y, Imai K, Kawai T, Onodera M. Persistent impairment of T-cell regeneration in a patient with activated PI3K δ syndrome. J Clin Immunol. 2017;37(4):347–50. 28432485 10.1007/s10875-017-0393-7

[111] Tsujita Y, Mitsui-Sekinaka K, Imai K, Yeh TW, Mitsuiki N, Asano T, et al. Phosphatase and tensin homolog (PTEN) mutation can cause activated phosphatidylinositol 3-kinase δ syndrome-like immunodeficiency. J Allergy Clin Immunol. 2016;138(6):1672–80.e10. 27426521 10.1016/j.jaci.2016.03.055

[112] Lougaris V, Baronio M, Moratto D, Tampella G, Gazzurelli L, Facchetti M, et al. A novel monoallelic gain of function mutation in p110δ causing atypical activated phosphoinositide 3-kinase δ syndrome (APDS-1). Clin Immunol. 2019;200:31–4. 30639166 10.1016/j.clim.2019.01.003

[113] Saettini F, Pelagatti MA, Sala D, Moratto D, Giliani S, Badolato R, et al. Early diagnosis of PI3Kδ syndrome in a 2 years old girl with recurrent otitis and enlarged spleen. Immunol Lett. 2017;190:279–81. 28842185 10.1016/j.imlet.2017.08.021

[114] Rivalta B, Amodio D, Giancotta C, Santilli V, Pacillo L, Zangari P, et al. Case report: successful treatment with monoclonal antibodies in one APDS patient with prolonged SARS-CoV-2 infection not responsive to previous lines of treatment. Front Immunol. 2022;13:891274. 35799775 10.3389/fimmu.2022.891274 PMC9253383

[115] Serra I, Manusama OR, Kaiser FMP, Floriano II, Wahl L, van der Zalm C, et al. Activated PI3Kδ syndrome, an immunodeficiency disorder, leads to sensorimotor deficits recapitulated in a murine model. Brain Behav Immun Health. 2021;18:100377. 34786564 10.1016/j.bbih.2021.100377 PMC8579111

[116] Wang Y, Chen X, Yang Q, Tang W, Jia Y, Zhou L, et al. E1021K homozygous mutation in PIK3CD leads to activated PI3K-delta syndrome 1. J Clin Immunol. 2020;40(2):378–87. 31953711 10.1007/s10875-020-00749-y

[117] Bucciol G, Willems L, Hauben E, Uyttebroeck A, Proesmans M, Meyts I. Thyroid carcinoma in a child with activated phosphoinositide 3-kinase δ syndrome: somatic effect of a germline mutation. J Clin Immunol. 2017;37(5):422–6. 28601916 10.1007/s10875-017-0407-5

[118] Lougaris V, Baronio M, Castagna A, Tessarin G, Rossi S, Gazzurelli L, et al. Paediatric MAS/HLH caused by a novel monoallelic activating mutation in p110δ. Clin Immunol. 2020;219:108543. 32681977 10.1016/j.clim.2020.108543

[119] Yagasaki H, Hirai M, Kanezawa K, Ueno M, Hao H, Masuda S, et al. Successful treatment for diffuse large B-cell lymphoma in a Japanese adolescent with PIK3CD germ-line mutation: stem cell transplantation after reduced-intensity conditioning. Ann Hematol. 2022;101(7):1617–9. 35247100 10.1007/s00277-022-04809-8

[120] Wang Y, Wang W, Liu L, Hou J, Ying W, Hui X, et al. Report of a Chinese cohort with activated phosphoinositide 3-kinase δ syndrome. J Clin Immunol. 2018;38(8):854–63. 30499059 10.1007/s10875-018-0568-x

[121] World Health Organization. Life tables: Life tables by WHO region Global. Accessed October 17, 2023. https://apps.who.int/gho/data/view.searo.LIFEREGIONGLOBAL

[122] Jamee M, Moniri S, Zaki-Dizaji M, Olbrich P, Yazdani R, Jadidi-Niaragh F, et al. Clinical, immunological, and genetic features in patients with activated PI3Kδ syndrome (APDS): a systematic review. Clin Rev Allergy Immunol. 2020;59(3):323–33. 31111319 10.1007/s12016-019-08738-9

[123] Carpier JM, Lucas CL. Epstein-Barr virus susceptibility in activated PI3Kδ syndrome (APDS) immunodeficiency. Front Immunol. 2017;8:2005. 29387064 10.3389/fimmu.2017.02005 PMC5776011

